# Contribution of Organic Food to the Diet in a Large Sample of French Adults (the NutriNet-Santé Cohort Study)

**DOI:** 10.3390/nu7105417

**Published:** 2015-10-21

**Authors:** Julia Baudry, Caroline Méjean, Benjamin Allès, Sandrine Péneau, Mathilde Touvier, Serge Hercberg, Denis Lairon, Pilar Galan, Emmanuelle Kesse-Guyot

**Affiliations:** 1 Equipe de Recherche en Epidémiologie Nutritionnelle, Centre de Recherche en Epidémiologie et Statistiques, Université Paris 13, Inserm (U1153), Inra (U1125), Cnam, COMUE Sorbonne Paris Cité, Bobigny F-93017, France; c.mejean@eren.smbh.univ-paris13.fr (C.M.); b.alles@eren.smbh.univ-paris13.fr (B.A.); s.peneau@eren.smbh.univ-paris13.fr (S.P.); m.touvier@eren.smbh.univ-paris13.fr (M.T.); s.hercberg@eren.smbh.univ-paris13.fr (S.H.); p.galan@eren.smbh.univ-paris13.fr (P.G.); e.kesse@eren.smbh.univ-paris13.fr (E.K.-G.); 2 Département de Santé Publique, Hôpital Avicenne, 125 rue de Stalingrad, Bobigny F-93017, France; 3 Department 'Nutrition, Obésité et Risque Thrombotique', Faculté de Médecine, Aix-Marseille Université, INSERM, UMR 1062, INRA 1260, Marseille 13385, France; denis.lairon@orange.fr

**Keywords:** organic, organic food consumption, dietary intakes, sustainable food

## Abstract

In developed countries, the demand for organic products continues to substantially increase each year. However, little information is available regarding the level of consumption of organic food and its relative share of the whole diet. Our aim was to provide, using individual consumption data, a detailed description of organic food consumption among French adults. Conventional and organic intakes were assessed using an organic food frequency questionnaire administered to 28,245 French adults participating in the NutriNet-Santé study. P values of Student *t*-test or Chi-square for the difference between genders were reported. Less than 12% of the respondents reported never consuming organic food in the past year. Women consumed on average 20% organic food in their whole diet per day while men consumed an average of 18%. The proportion of vegetables consumed that came from organic sources was 31% among women and 28% among men. Overall, the estimate of the contribution of organic food from products of plant origin was higher than that from products of animal origin. Our study provides a framework for the exploration of organic consumption and its correlates and can serve as a basis for future studies investigating relationships between the level of organic food consumption and health outcomes.

## 1. Introduction

It is now widely recognized that current food patterns are unsustainable over the long term [1,2]. It seems necessary to meet the growing demand for food in a manner that is ecologically sustainable. Due to its reduced environmental impact, organic farming might be considered as a potential alternative to intensive industrial agriculture [3,4,5,6,7]. Moreover, with ethical considerations, one of the main reasons for organic food consumption appears to be the alleged beneficial effects on human health [8,9,10]. Nevertheless, strong evidence is lacking concerning nutritional differences between organic and conventional foods [11,12,13,14,15]. Moreover, few studies have investigated the direct impacts of the type of farming on health [16,17,18,19,20]. In a recent, large prospective study [17], it has been shown that there is little or no decrease in the incidence of cancer associated with the consumption of organic food, except possibly for non-Hodgkin lymphoma. However, organic food consumption was assessed using a relatively simple questionnaire and no information about the type of food consumed was collected. It is therefore necessary to go further in the analysis of the organic food diet and the type of food consumed to better understand the potential health effects of such a diet.

In this context, the worldwide organic food market has increased more than four-fold in 12 years, reaching 55 billion euros in 2013 [21]. The European market for organic products was valued at approximately 24.3 billion euros in 2013 [21]. The French organic market, valued at almost 5 billion euros [22], is the second largest in Europe after Germany and before Italy and the third largest organic market in the world [21,22,23]. However, despite this tremendous increase in the past decade, the organic food market remains modest throughout France, representing only 2.6% of the food market in 2014 [22].

According to the French Organic Agency [24], the market share of organic products varies across sectors. In 2013, more than half of the organic sales were fresh products. Thus, this share was 20% for eggs and 10% for milk [24]. This value represented 6% for the 14 most consumed fruits and vegetables (excluding citrus and bananas) while it represented between 2% and 3% for beef and pork meats and only 0.5% for processed meat [24]. That same year, 75% of organic products consumed in France came from France and, among the products imported from other countries, 44% were exotic products such as coffee, tea and chocolate [24].

Besides, according to a report released in 2015 by the same French Agency, 62% of French consumers claim to consume organic food at least once a month [10].

Nevertheless, there is little information available about the place organic food holds in total food intake and its importance according to food groups. Previous studies tend to focus on the frequency of organic food consumption or purchase [25,26,27,28] and few studies [29], none in Europe, examine the quantities of organic food consumed as a percentage of the overall diet. It has been shown that women are more inclined to purchase organic food for the household [30,31] than men**.** However, little is known about the actual consumption of organic food, in particular with a high level of precision, across genders.

It seems, therefore, crucial to describe the level of organic consumption to better assess health impacts of organic foods according to their contribution to the overall diet and to focus on dose-dependence.

This study aims to provide detailed information about organic food consumption from individual data collected among a large sample of French adults and to give an overall description of the level of organic food consumption, its relative share in the whole diet and the specificities pertaining to individual foods and food groups, as well as the percentages of organic food consumers of each food group.

## 2. Experimental Section

### 2.1. Ethics

The present study was conducted according to the guidelines laid down in the Declaration of Helsinki. The NutriNet-Santé study was approved by the Institutional Review Board of the French Institute for Health and Medical Research (IRB Inserm no. 0000388FWA00005831) and the “Commission Nationale de l'Informatique et des Libertés” (CNIL no. 908450 and no. 909216). All subjects signed an electronic informed consent. This study is registered in EudraCT (n2013-000929-31).

### 2.2. Participants

The NutriNet-Santé Study was launched in May 2009 in France with a scheduled follow-up of at least 10 years. It is an ongoing, web-based, prospective observational cohort which aims at investigating the relationship between nutrition and health as well as the determinants of dietary patterns and nutritional status. The design and methodology of the NutriNet-Santé study have been described in detail elsewhere [32].

### 2.3. Data Collection

#### 2.3.1. Assessment of Individual Characteristics

Participants filled in self-administrated questionnaires using a dedicated website at baseline and at different months of follow-up. The baseline questionnaires were pilot-tested and then compared against traditional assessment methods [33,34]. These questionnaires were used to regularly collect data on demographic, socioeconomic and lifestyle characteristics, including age, gender, smoking status, physical activity (as measured by the IPAQ [35]), geographical region, marital status, number of children, educational level, socio-professional category and level of income. Income per household unit was calculated using information about household income and composition. Household income per month was divided by the number of consumption units (CU) calculated: 1 CU for the first adult in the household, 0.5 CU for other persons aged 14 or older and 0.3 CU for children under 14 [36]. Current practices of diets (type and reason, history) were also collected [37]. In particular, subjects were asked whether they were following a vegan or a vegetarian diet. A vegetarian diet was defined as a diet that did not include any meat while a vegan diet was defined as a diet that excluded all products of animal origin.

#### 2.3.2. Organic Food Frequency Questionnaire: Org-FFQ

Initially, a semi-quantitative food frequency questionnaire (FFQ) was used in the NutriNet-Santé Study for self-administered assessment of usual dietary intake over the past year among French adults. The reproducibility and relative validity of this FFQ were previously tested against 24-hour dietary records (DRs) and acceptable reproducibility and relative validity were observed [38].

The volunteers were asked to report their consumption frequencies for 264 food and beverage items over the past year. The 264 items were divided into main food group categories. Additional questions inquired about the types of butter and margarine used for frying and baking and on bread. For most food items, subjects were asked to report their consumption frequency on the basis of how many times they ate the standard portion size proposed (typical household measurements such as spoon or standard unit such as a yogurt). The frequency of consumption referred to usual consumption over the past year on an increasing scale including yearly, monthly, weekly or daily units, as suitable, and participants were asked to provide only one answer.

**Figure 1:**
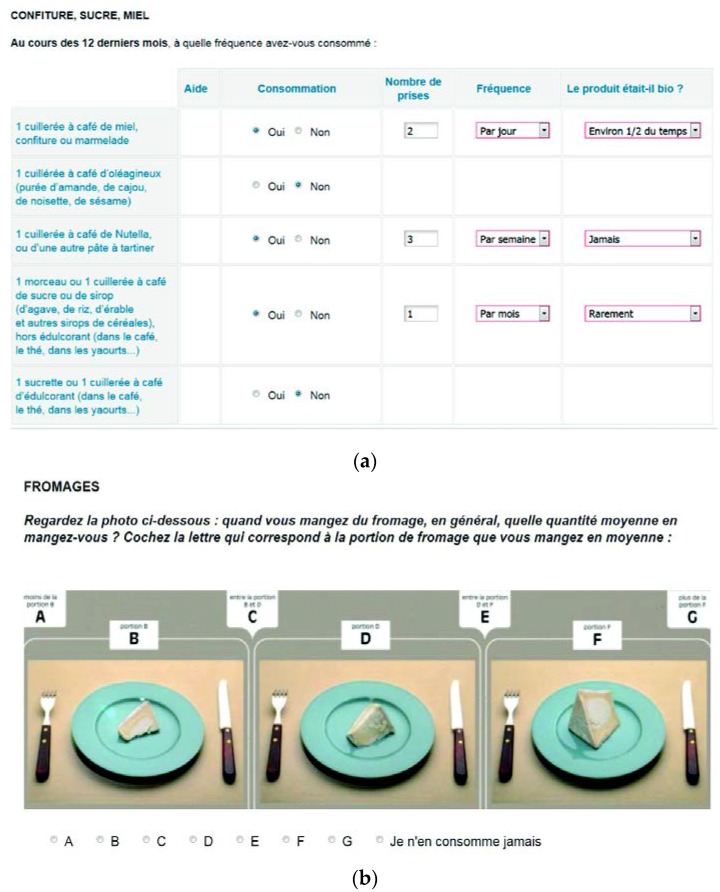
Extracts of the Org-FFQ, NutriNet-Santé Study, *N* = 28,245; (**a**) For each food item consumed, the quantity, the frequency of the intake and the relative organic share were asked; (**b**) For eight categories which are usually not eaten in a predetermined portion size, the questionnaire included sets of colour photographs.

For eight of the main food group categories (cheese and vegan cheese, pâté and vegan pâté, fish, meat, butter used on bread, potatoes, starchy foods and vegetables), which are usually not eaten in a predetermined portion size, the questionnaire included sets of colour photographs. Participants were asked to choose among three photographs showing different portion sizes. Together with the two intermediate and two extreme quantities, seven choices of amounts were therefore possible. For butter on a slice of bread, four portion sizes were proposed. These photographs had been previously validated [37] (Figure 1). Standard portion sizes or portion size corresponding to the photographs were multiplied by the daily frequencies to estimate the intake of each food item in grams.

Based on this original FFQ, the organic food frequency questionnaire (Org-FFQ) was developed. For each food item, except those that do not exist in organic form (*i.e.*, water and sweetener products) a 5-point ordinal scale ranging from “never” to “always” was used to determine the proportion of intake that was of organic origin. Participants were asked to answer the following question: “How often was the product of organic origin?” For butter and margarine used for bread and frying, participants were asked to choose the most frequently consumed item among approximately 20 organic or conventional items. To estimate the organic intake, for each food item, a weight of 0, 0.25, 0.5, 0.75 and 1 was respectively applied to the following modalities: never, rarely, half the time, often and always. In order to better understand the impact of allocating arbitrary percentages, sensitive analyses were performed. A percentage of 10% instead of 25% was allocated to the modality rarely. Furthermore, 20 Monte-Carlo simulations were also performed [39]. For this purpose, to each category of frequency (never, rarely, half-of-the-time, often and always), arbitrary intervals were assigned as follows: the “never” modality was equivalent to a frequency comprising between 0% and 2.5%, rarely between 2.5% and 35%, half of the time between 35% and 65%, often between 65% and 90%, and always between 90% and 100%. It was hypothesized that the modalities were uniformly distributed within those intervals. For one set of data, the same percentage was attributed to one particular modality. Although the food frequency questionnaire used showed acceptable reproducibility and relative validity, the question relating to the frequency of organic food consumption was not validated.

### 2.4. Statistical Analysis

From the 264 food items, 33 food groups were developed on a nutritional basis. Because of the high contribution of beverages in terms of weight to the total intake, we distinguished liquid products from solid ones. In all individuals, we calculated the average quantity (in g/day) of the whole diet, the solid-based diet, the liquid-based diet and the average quantity consumed for the 33 food groups. This was performed for the overall diet and for the organic food diet. In a second step, we assessed the average proportion of organic food consumed in the whole diet, the liquid-based diet, the solid-based diet, and by food group among consumers of each food group, namely the ratio for these indicators.

In a final step, we determined the 10 most popular organic food items according to several criteria. In a first approach, we defined this top 10 in terms of number of consumers. The top 10 organic foods were also calculated in terms of absolute organic intake (g/day) and relative intake. For each food item, the relative organic intake was calculated by averaging the total organic food intake (g/day) out of the total intake (g/day). Finally, in all individuals, we calculated the most frequently consumed organic items by multiplying the daily frequency of consumption by the frequency of consumption in its organic form.

In all individuals and among organic consumers (*i.e.*, consumers who did at least report consuming one organic food item) the following was also calculated: the percentage of subjects who consumed each food group, the percentage of consumers having at least 50% of the food group of organic origin and the percentage of consumers having 100% of the food group of organic origin.

The average total and organic food consumptions (g/day) were also examined for the overall diet and by food groups according to age, formal education, income, location, physical activity and type of diet (meat-eaters *vs.* vegetarians and vegans). The share of organic food consumption in the diet was also calculated according to these factors and means, standard deviations and medians were provided.

The Org-FFQ was administered over a 5-month period from June to October 2014. A total of 33,384 persons had completed the Org-FFQ. Only participants with a plausible energy intake were included in the analyses for dietary intakes to avoid unrealistic estimates as diet underreporting and overreporting participants were identified. Briefly, basal metabolic rate (BMR) was estimated by Schofield equations [40] according to gender, age, weight and height collected at enrollment in the study. Energy requirement, accounting for physical activity level (set by default at 1.55) and BMR, was compared with energy intake. The ratio between energy intake and energy requirement was calculated and individuals with ratios below or above cutoffs previously identified (0.35 and 1.93) in the FFQ were excluded. Thus, we excluded 2097 individuals with inappropriate energy intake, 2320 individuals with missing covariates and 722 participants residing in overseas territories, thus leaving 28,745 participants available for analysis (20,980 women and 7265 men).

For each gender, weighting was calculated using the iterative proportional fitting procedure according to 2009 national census reports [41] on age, occupational category, area of residence and whether or not the household included at least one child (<18 years).

We compared the socio-demographic characteristics of included and excluded NutriNet-Santé participants using chi-square tests and Student *t*-tests, as appropriate. Due to well-known differences in dietary patterns across genders, all analyses were performed separately for women and for men.

Descriptive characteristics of the sample are presented as means (± SD) or *n*% as appropriate and *p* values of Student *t*-test or Chi square for the difference between genders are reported.

Tests of statistical significance were 2-sided and the type I error was set at 5%. Statistical analyses were performed using SAS software (version 9.3, SAS Institute Inc., Cary, NC, USA).

All the results presented are weighted data.

## 3. Results

### 3.1. Description of the Study Population

To better understand the selected sample, we compared characteristics of individuals who completed the questionnaire (*N* = 33,384) (before weighting) and excluded NutriNet-Santé participants (*N* = 123,239). The percentage of women who completed the Org-FFQ was lower (74% *vs.* 79%), the respondents were also more likely to be retired (36% *vs.* 14%), older (53.20 ± 14.07 year *vs.* 44.62 ± 14.20 year) and more often a holder of a master degree (34% *vs.* 33%).

Sociodemographic and lifestyle characteristics by gender are summarized in Table 1.

**Table 1:** Sociodemographic and lifestyle characteristics of the weighted sample, NutriNet-Santé Study, *N* = 28,245.

	Women		Men		*p* *
	Mean or %	SD	Mean or %	SD	
***N*—not weighted %**	20,980 (74.28)		7265 (25.72)		
***N*—weighted %**	14,788.40 (47.64)		13,456.60 (52.36)		
**Age (years)**	48.65	13.88	47.39	21.92	**<0.0001**
**Educational level**					**<0.0001**
<High school diploma	58.39		61.25		
High school	16.07		14.56		
Post-secondary graduate	25.54		24.19		
**Income per household unit ^†^**					**<0.0001**
<1.200 euros	24.68		23.85		
1.200–1800 euros	25.50		24.17		
1.800–2.700 euros	22.04		24.72		
>2700 euros	10.66		15.83		
Missing	17.12		11.43		
**Socio-professional categories**					**<0.0001**
Farmer	0.55		1.53		
Craftsman, shopkeeper, business owner	1.87		5.14		
Non employed	7.74		1.32		
Employee	25.31		8.41		
Student	4.60		4.40		
Manual worker	5.22		23.49		
Intermediate profession	14.47		14.53		
Retired	28.61		26.12		
Managerial staff	4.89		3.37		
**Location**					**<0.0001**
Rural community	24.60		26.23		
Urban unit with a population smaller than 20,000 inhabitants	15.32		15.13		
Urban unit with a population between 20,000 and 200,000 inhabitants	16.22		16.85		
Urban unit with a population higher than 200,000 inhabitants	43.86		41.79		
**Smoking status**					**<0.0001**
Never smoker	52.66		41.56		
Former smoker	33.78		42.94		
Current smoker	13.56		15.50		
**Physical activity**					**<0.0001**
High	20.92		16.83		
Medium	34.02		29.20		
Low	31.58		37.87		
Missing	13.48		16.10		
**Vegetarian diet**	2.88		1.53		**<0.0001**
**Vegan diet**	3.25		2.93		0.12
**Location**					**<0.0001**
Rural community	24.60		26.23		
Urban unit with a population smaller than 20,000 inhabitants	15.32		15.13		
Urban unit with a population between 20,000 and 200,000 inhabitants	16.22		16.85		
Urban unit with a population higher than 200,000 inhabitants	43.86		41.79		

* *p*-values based on Student *t*-test or Chi squared for difference between genders as appropriate; ^†^ By consumption unit in the household: official weighting system by the French National Institute of Statistics and Economic Studies INSEE; SD, standard deviation.

Compared to women, men were younger while they were more likely to present a post-graduate formal education level, an income per household unit of >2700 euros, and a low physical activity level. They were also more likely to be managerial staff, single and smokers. The percentage of individuals following a vegetarian or vegan diet was higher among women than among men.

### 3.2. Percentage of Organic Food Consumers by Food Group

The Table S1 provides, in the entire sample and among organic food consumers, (1) the percentage of consumers of each food group; (2) the percentage of consumers having at least 50% of the food group with organic origin and (3) the percentage of consumers having 100% of the food group with organic origin.

The percentage of non-organic food consumers (*i.e.*, individuals consuming 0 g/day of organic foods) was 8.4% in women and 14.7% in men. Vegetables, fruits, cereals and sweetened products were largely consumed by the participants and by organic food consumers in particular, with percentages of consumers higher than 98%. More than a quarter of organic food consumers reported eating at least 50% of vegetables, fruits and related products of organic sources. Milk was consumed by only 35.6% of the study population but 24.4% of the consumers reported consuming at least 50% of their milk from organic sources, while among the 89.4% subjects who consumed dairy products this percentage did not reach 20%.

### 3.3. Contribution of Organic Food to the Whole Diet by Gender

Table 2 shows the relative contribution of organic food in the whole diet in terms of weight and energy by gender.

As expected, compared to women, men had a higher total intake but a lower intake from organic sources. Women consumed on average 695.62 ± 673.35 g/day of organic food, and men consumed 621.79 ± 1002.10 g/day. The proportion of organic sources in the diet was significantly different across genders: organic foods contributed to 20% to the whole diet among women and 18% among (*p* < 0.0001). When excluding the liquid products and the water in particular, corresponding proportions were 28% among women and 25% among men.

### 3.4. Contribution of Organic Food to Food Groups

Results in Table 3 describe the mean intake of 33 food groups (overall and organic). Compared to women, men had higher total food intakes of fruit juices, products of animal origin (meat, processed meat, poultry, eggs, milk and cheese), starchy food, sugary products, alcoholic beverages and soda.

Concerning the contribution of organic food to different food groups, women consumed a significantly higher proportion of organic foods for most food groups compared with men except for processed meat, fish, cheese, alcohol, dairy substitutes and soda. Among women, the contribution of organic food to the total intake ranged from 0.11 ± 0.14 (non-alcoholic drinks) to 0.81 ± 0.26 (meat substitutes) whereas among men, ratios ranged from 0.09 ± 0.20 (non-alcoholic drinks) to 0.76 ± 0.52 (meat substitutes). In both genders, the proportion of fruits and vegetables consumed that came from organic sources was around one-third while this contribution was less than 20% for meat and fish and around one quarter for cereals.

Table S2 shows a comparison between the contributions of organic food to the diet and by food group across gender using a fixed percentage of 25% for the modality rarely, a fixed percentage of 10% for the modality rarely and using Monte-Carlo simulations to affect percentages to each modality.

Attributing a frequency of 10% to rarely did not change substantially the results (−2.5% on average) while the impact of the Monte-Carlo simulations was even lower (−1% on average). The share of organic food in the whole diet was 18% among women and 16% among men when allocating a percentage of 10% to rarely while it was 19% among women and 17% among men when using Monte-Carlo simulations.

Overall, older subjects (*i.e.*, above the median value equals to 48 years old), individuals with high school diploma, with an income per household unit higher than 1800 euros per month, living in rural area, undertaking physical activity of more than 30 min of brisk walking per day, and vegetarians/vegans demonstrated higher intake of organic food (g/day) than their counterparts (Table S3).

### 3.5. Top 10 Organic Food Items

The top 10 most consumed organic food items in terms of number of consumers, of absolute intake (g/day), of relative organic food intake and of frequency per day are presented in Table 4 for women and men.

In terms of number of consumers, eggs were the products consumed by the largest number of individuals in their organic form among both genders out of the 264 food items. Among the 10 most commonly consumed items (as regards quantity in g/day), six of them (apple, green salad, tomato, citrus fruit, cucumber and peach) in women and five of them in men (apple, green salad, tomato, banana, carrot) were fruits and vegetables. Organic whole bread was also largely consumed in both genders (it held the fourth place in women and the third in men). The food items with the highest organic food contributions were specific foodstuffs rarely consumed by participants in general. Thereby, only a weighted number of “573” women reported eating linseed oil while a weighted number of “695” men reported eating seitan. The food item “honey/jam” was the most frequently consumed item in its organic form in both genders, since this product was consumed in its organic form more than every three days.

### 3.6. Contribution of Organic Food to the Whole Diet According to Several Sociodemographic and Lifestyle Factors

Table 5 provides the share of organic food in the whole diet according to several factors of variation: gender, age, education, income, location, physical activity and type of diet.

**Table 2:** Share of organic food in the whole diet by gender (g/day and kcal/day), NutriNet-Santé Study, *N* = 28,245.

	Women						Men						*p* *
	*n* = 20,980						*n* = 7265						
	Total		Organic		Ratio		Total		Organic		Ratio		
	Mean	SD	Mean	SD	Mean	SD	Mean	SD	Mean	SD	Mean	SD	
Total Intake (g/day)	3408.34	949.91	695.62	673.35	0.20	0.18	3497.16	1666.2	621.79	1002.10	0.18	0.28	**<0.0001**
Liquid intake (g/day) ^†^	1108.00	461.83	290.20	328.16	0.26	0.25	1130.69	825.85	255.77	499.49	0.23	0.40	**<0.0001**
Solid intake (g/day) ^‡^	1360.44	478.29	405.42	425.18	0.28	0.23	1396.72	812.46	366.02	624.75	0.25	0.38	**<0.0001**
Water intake (g/day)	939.90	44.34	NA	NA	NA	NA	969.75	982.85	NA	NA	NA	NA	/
Total intake (kcal/day)	1979.06	539.19	537.74	502.91	0.27	0.23	2280.27	1016.5	546.54	852.48	0.24	0.36	**<0.0001**
Liquid intake (kcal/day) ^†^	198.16	121.83	53.17	70.74	0.27	0.25	246.58	248.59	56.71	125.54	0.23	0.39	**<0.0001**
Solid intake (kcal/day) ^‡^	1780.90	05.72	484.57	460.07	0.27	0.23	2033.69	955.08	489.83	776.98	0.24	0.36	**<0.0001**

* *p*-values based on Student *t*-test (for difference between ratios); ^†^ Liquid products (including soups and beverages); ^‡^ Solid products; SD, standard deviation; NA, not applicable.

**Table 3:** Share of organic food by food groups by gender (g/day), NutriNet-Santé Study, *N* = 28,245.

Food groups	Women								Men								*p* ^§^
	*n* = 20,980								*n* = 7265								
	*N* *	*N* ^†^	Total		Organic		Ratio ^‡^		*N* *	*N* ^†^	Total		Organic		Ratio ^‡^		
			Mean	SD	Mean	SD	Mean	SD			Mean	SD	Mean	SD	Mean	SD	
Vegetables	20962	14778.38	277.93	187.84	107.36	158.41	0.31	0.27	7253	13443.91	254.34	260.31	83.43	185.50	0.28	0.44	**<0.0001**
Soup	19697	13327.17	84.52	89.28	34.12	58.14	0.34	0.30	6426	10818.15	54.22	123.81	19.24	71.63	0.33	0.46	**0.0017**
Fruits	20925	14710.15	320.03	274.75	99.73	148.55	0.29	0.26	7241	13212.14	249.30	389.33	75.42	228.72	0.28	0.44	**0.0102**
Fruit juice	16865	11268.17	84.66	95.29	27.53	49.92	0.33	0.28	5803	10418.84	87.40	161.38	28.78	95.16	0.29	0.46	**<0.0001**
Nuts	15781	10032.06	3.59	6.54	1.78	4.44	0.35	0.30	5178	8248.876	2.42	8.35	1.15	5.88	0.33	0.48	**0.0007**
Meat	19801	13404.12	57.16	48.26	9.49	16.72	0.18	0.22	7013	12613.52	88.87	143.19	14.25	39.64	0.18	0.35	0.9520
Processed meat	19694	13236.63	29.48	24.09	4.06	7.60	0.15	0.19	6973	12375.27	40.05	48.38	6.48	19.82	0.16	0.32	**0.0074**
Fish	20161	13622.62	41.56	36.46	6.32	12.24	0.15	0.19	6994	11930.79	40.75	63.88	7.44	27.53	0.16	0.33	**0.0015**
Poultry	19849	13437.17	23.44	22.41	5.50	8.72	0.27	0.26	6964	12537.7	27.95	43.61	6.35	15.22	0.26	0.42	**0.0006**
Eggs	20095	13743.7	11.40	10.11	6.17	7.83	0.52	0.34	6972	12698.54	14.17	40.41	5.14	14.58	0.40	0.54	**<0.0001**
Milk	6895	5038.406	63.76	117.71	15.32	53.17	0.29	0.32	2276	5015.25	67.56	196.43	17.29	94.05	0.28	0.56	0.3421
Dairy products	19444	13286.39	162.75	129.79	33.94	56.95	0.24	0.27	6551	11963.28	134.93	173.31	25.89	72.39	0.23	0.44	**0.0015**
Cheese	20283	13936.1	36.81	35.31	5.15	10.53	0.15	0.20	7009	12699.95	46.03	65.88	7.63	23.52	0.17	0.33	**<0.0001**
Milky desserts	15110	9678.505	12.02	23.16	1.56	7.47	0.14	0.21	4994	8301.999	11.99	31.79	1.10	6.20	0.12	0.30	**<0.0001**
Potatoes	20773	14660.22	21.32	18.13	6.15	11.12	0.28	0.30	7216	13266.06	35.44	65.71	8.36	19.84	0.26	0.46	**0.0001**
Bread	17942	12361.84	46.53	44.39	6.00	13.22	0.16	0.21	6118	11568.34	67.80	86.71	9.23	31.26	0.16	0.34	0.1516
Cereals **^|^**^|^	20844	14673.23	77.51	59.03	20.58	34.06	0.28	0.29	7184	13386.24	112.95	140.97	25.73	70.35	0.24	0.45	**<0.0001**
Wholegrain products ^¶^	17489	11521.01	58.28	73.68	28.03	52.53	0.37	0.30	5521	9193.965	52.27	113.79	28.01	95.61	0.37	0.48	0.7229
Oil	20744	14489.61	19.86	14.22	7.99	10.91	0.36	0.32	7146	12882.69	17.21	26.80	6.42	14.90	0.33	0.51	**<0.0001**
Cookies	19032	13175.31	11.11	17.47	1.24	3.73	0.15	0.22	6512	11538.17	14.10	28.95	1.46	6.58	0.13	0.31	**<0.0001**
Nonalcoholic drinks **	20978	14786.7	1680.03	708.18	182.76	256.34	0.11	0.14	7262	13454.84	1668.14	1220.4	143.17	358.47	0.09	0.20	**<0.0001**
Sweet	20925	14743.73	46.51	35.03	11.66	19.98	0.25	0.24	7241	13229.96	54.53	59.74	12.38	25.75	0.24	0.38	**0.0238**
Fast food	20371	14204.8	31.03	25.10	5.05	9.42	0.17	0.22	7050	12767.22	65.60	311.61	11.65	78.03	0.15	0.32	**<0.0001**
Meat substitutes	6796	4428.214	7.94	21.50	7.02	19.74	0.81	0.26	1654	3306.038	6.24	35.34	5.17	32.39	0.76	0.52	**<0.0001**
Dressing	20489	14275.82	7.15	6.92	1.50	3.57	0.22	0.26	7088	12758.5	7.69	11.97	1.42	5.09	0.20	0.39	**<0.0001**
Alcohol	19069	12420.19	60.02	81.41	9.16	25.87	0.14	0.18	6892	12177.21	136.47	256.24	20.95	72.25	0.16	0.31	**<0.0001**
Snacks	19666	13358.92	9.91	11.92	3.13	7.96	0.19	0.25	6798	12325.1	10.00	24.07	2.47	11.48	0.17	0.37	**<0.0001**
Grains	9325	5780.877	3.70	8.19	2.90	7.03	0.70	0.31	2279	4429.612	2.12	9.56	1.63	8.19	0.64	0.55	**<0.0001**
Other fats ^††^	19798	13777.13	4.01	5.48	1.00	2.38	0.25	0.29	6521	11510.38	2.79	5.70	0.83	3.49	0.23	0.43	**<0.0001**
Dairy substitutes ^‡‡^	6170	3992.64	30.90	81.47	25.10	73.05	0.63	0.33	1487	2864.714	30.40	157.46	25.40	146.82	0.65	0.58	0.1721
Legumes	18809	12558.85	20.21	42.80	12.18	41.06	0.31	0.31	6521	11104	20.53	48.81	9.04	41.63	0.28	0.47	**<0.0001**
Soda	14716	10210.88	56.59	117.15	4.72	23.04	0.11	0.20	4919	10124.65	66.25	206.90	7.44	42.63	0.13	0.36	**<0.0001**

* Number of consumers of each food group (not weighted); ^†^ Number of consumers of each food group (weighted); ^‡^ Ratio calculated among consumers of each food group; ^§^ *p*-values based on Student *t*-test (for difference between ratios); **^|^**^|^ Including pasta, white rice, muesli, semolina and breakfast cereals; ^¶^ Including wholegrain bread, wholegrain rice and wholegrain pasta; ** Including coffee, tea, chicory, hot chocolate and water; ^††^ Including mayonnaise, fresh cream, vegetal fresh cream; ^‡‡^ Including soy yogurt, vegetal-based cheese, vegan fresh cheese, soy milk.

**Table 4:** Top 10 Organic Food items by gender, NutriNet-Santé Study, *N* = 28,245.

	In Terms of Number of Consumers	*N* *	In Terms of Weight ^†^,^‡^	g/day	In Terms of Contribution in the Intake ^§^	%	*N* *	In Terms of Frequency per day ^†^	
**Women *n* = 20,980**									
1	fried eggs	9192	apple	23.31	linseed oil	92	573	honey, jam	0.35
2	hard boiled eggs	8754	green salad	20.02	kombucha	91	179	olive oil	0.33
3	tomato	8655	tomato	17.08	vegan chorizo	90	626	tea	0.27
4	honey, jam	8328	whole bread	13.91	soy milk	90	1979	whole bread	0.25
5	cucumber	8096	legumes	12.18	vegan pâté	89	1111	herbal tea	0.20
6	olive oil	7949	citrus fruit	11.51	vegan fresh cheese	89	14089	green salad	0.20
7	green salad	7948	full fat yoghurt	11.01	seitan	89	831	chocolate	0.17
8	strawberry	7775	cucumber	10.3	vegan galette	88	2636	black coffee	0.16
9	peas	7719	peach	9.71	sprouted seeds	87	1332	tomato	0.16
10	apple	7715	whole rice	9.46	safflower oil	87	134	spreadable butter	0.15
**Men *n* = 7265**									
1	fried eggs	7367	apple	21.16	seitan	98	695	honey, jam	0.39
2	tomato	7215	green salad	15.28	vegan fresh cheese	98	341	whole bread	0.28
3	honey, jam	6889	whole bread	15.14	coconut oil	97	360	olive oil	0.27
4	olive oil	6788	tomato	13.96	vegan pâté	96	981	black coffee	0.22
5	apple	6570	banana	9.99	vegetal-based cheese	95	254	chocolate	0.19
6	green salad	6500	legumes	9.04	kombucha	94	369	tea	0.18
7	strawberry	6477	white bread	8.74	linseed oil	93	427	white bread	0.16
8	peas	6452	pasta	8.32	soy-based cheese	92	225	green salad	0.14
9	cucumber	6429	full fat yoghurt	8.25	vegan chorizo	91	408	spreadable butter	0.14
10	carrot	6216	carrot	7.98	vegan galette	89	1349	apple	0.13

* Weighted number of consumers of each food item; ^†^ in all individuals; ^‡^ only solid products were considered; ^§^ among consumers of each food item.

**Table 5:** Share of organic food in the whole diet according to several sociodemographic and lifestyle factors, NutriNet-Santé Study, *N* = 28,245.

	Weighted %	Ratio			*p* *
		Mean	SD	Median	
**Gender**					<0.0001
Women	47.64	0.20	0.18	0.14	
Men	52.36	0.18	0.28	0.10	
**Age**					<0.0001
≤Median age (48 years old)	50.10	0.18	0.25	0.11	
>Median age (48 years old)	49.90	0.19	0.18	0.13	
**Education**					<0.0001
<High school diploma	75.11	0.18	0.30	0.10	
≥High school diploma	24.89	0.21	0.13	0.16	
**Income**					<0.0001
<1800 euros	57.43	0.17	0.25	0.09	
≥1800 euros	42.57	0.22	0.18	0.16	
**Location**					<0.0001
Rural area	25.44	0.20	0.22	0.13	
Urban area (community ≥ 5000 inhabitants)	74.56	0.19	0.20	0.12	
**Physical activity**					<0.0001
<30 min brisk walking/day	22.25	0.14	0.18	0.07	
≥30 min brisk walking/day	77.75	0.21	0.21	0.16	
**Type of diet**					<0.0001
Meat eaters	94.67	0.17	0.19	0.11	
Vegetarians and vegans	5.33	0.47	0.29	0.48	

* *p*-values based on Student *t*-test (for difference between ratios).

Individuals older than 48 years old, with high educational level and income, living in rural area and undertaking medium or high physical activity as well as those who followed a vegan or vegetarian diet had higher contributions of organic food in their intake than their counterparts. The consumption of organic food of half of the vegetarians and vegans constituted more than 48% of their diet, while 50% of the meat eaters had a diet consisting of less than 11% of organic food.

## 4. Discussion

The current study provides a detailed description of organic food consumption in the whole diet in a large French adult population from the NutriNet-Santé study. Less than 12% of the respondents reported never consuming organic food over the past year. Women consumed on average 20% of organic food in their whole diet per day while men consumed an average of 18%. The proportion of vegetables consumed that came from organic sources was 31% among women and 28% among men and for eggs was 52% among women and 40% among men. These contributions were less than 20% for meat and fish and around one quarter for cereals.

In the current study, we found that the percentage of non-consumers was equal to 11.4%. This was found to be consistent with the survey question asked by the French Organic Agency “*Have you consumed any organic products over the past year?*” where 12% of the 506 respondents reported never consuming any organic foods. We also found that the percentage of individuals that had reported never consuming any organic foods was lower among women (8.4%) than among men (14.7%). This finding was in accordance with the results of the same survey where 10% of women and 15% of men had reported never consuming organic food [10]. Another noteworthy finding of our study was that the highest consumption of organic food was by women compared to men in terms of absolute intake and in terms of relative share of organic consumption in the diet. This relative share was significantly higher in women than in men (*p* < 0.0001). These results seem consistent with other studies showing that women are more willing to pay than men for organic food mainly for health considerations [31,42]. In accordance with previous works [28,43,44,45,46], the current study also found that individuals with a high education level, a higher level of physical activity and following a vegetarian or vegan diet had a higher contribution of organic food in their diet compared to their counterparts. Surprisingly, the relative share of organic food in the diet was higher among individuals living in rural areas than those living in urban areas, contradicting previous research [27,45].

To our knowledge, there was only one study which investigated the contribution of organic food to the diet. That study focused on regular organic consumers and was conducted in Australia on a limited number of participants [29]. In their study, two questionnaires were administered to participants; the amount of organic food consumption was calculated based on quantification of serving size by food group among 19 participants. The frequency of organic consumption was also examined for nine food groups (*N* = 318). Unsurprisingly, as the survey targeted regular consumers of organic foods, higher percentages were obtained for organic food consumption from their study. The study conducted using three 24 h records from 19 participants, found that the percentage of their diet that came from organic sources based on the relative amount in diet was 76.3% from organic sources which compares with 20% in women and 18% in men in our study.

In our study, another important finding was that overall the contribution of organic food to the diet was higher for products of plant origin than for products of animal origin. The only exception was eggs: 52% of the consumption of eggs was of organic origin for women and 40% for men. Eggs were the top food from an animal source consumed in organic form among the top 10 organic foods. In regards to organic animal products’ consumption, organic eggs were followed by organic milk. Our findings were in accordance, for some food groups, with the previously cited Australian study [29]. Thus, according to this study, the most popular organic food groups were fruits and vegetables and the least popular were meat products (including poultry and fish) [29].

In our study, 27.2% of the subjects ate more than half of their vegetables from organic sources and 24.4% of fruit consumers ate more than half of their fruit in organic form. These results are consistent with the survey of the French Organic Agency in which 28% of the respondents ate more than half of their fruits and vegetables in their organic form [10]. It has been found that organic food consumers tend to be large consumers of fruits and vegetable [25,27,28], and in the past decade, the share of land dedicated to organic fruits and vegetables has increased. Thus, the share of organic farms dedicated to fruits and vegetables was found to be 16% while 8% of the farms from all types of production (conventional and organic) in France in 2013 were organic [24].

Regarding meat and fish, the contribution of organic foods to these food groups was lower than 20%. These results may seem high compared to the share of the organic meat in the market which represents 0.77% (pig farming) to 7% (laying hens) of the sector [24]. Nevertheless, they fall within the framework of an increase of organic meat production while overall a decrease of meat consumption is observed (−2.5% in 2013) [47]. Along with the high contribution of organic eggs in egg consumption, organic poultry was found to make a relatively high contribution in the consumption of poultry (27% and 26% in women and men, respectively) compared to other meat products. Consistent with the survey by the French Organic agency, the percentage of consumers having 100% of poultry from organic sources was 6.7% (*vs.* 5% in the survey) [10]. The relatively low consumption of organic fish was expected given that this foodstuff is mostly available in its conventional form. The sector of organic seafood represented only 1.1% of the market in 2013. Moreover, wild fish do not exist in organic form and organic fish in France are mostly imported [24].

Dairy products and cheese were food groups largely consumed by the study population (consumed by around 90% of participants) unlike milk which was only consumed by 35.6% of the subjects. Nevertheless, the contribution of organic foods to these food groups (around one quarter for dairy products and around 15% for cheese) was lower in comparison to milk (around 30%). When comparing with the market share of organic foods, which represented more than 10% of the milk market in 2013 in France, these results may seem high [24]. Nevertheless, these results follow the trend of the global milk and related product market [48]. Thus, unlike the conventional sector in which milk represented only 10% of the transformations, packaged milk retained a prominent place in the organic sector. However, the trend is reversed for cheeses (11% of the transformations) while they represented 37% of conventional milk transformations [48].

Among both women and men, on average, 37% of the consumption of wholegrain products came from organic sources. This high proportion may be explained by the fact that organic wholegrain products are quite common and largely consumed by organic food consumers [25]. The highest contributions of organic foods (with ratios higher than 0.60 for both men and women) were meat- and dairy-substitutes. This was not surprising as the main part of these foodstuffs is consumed in organic form. This can be explained by the broad range of organic offerings and the fact that some of these products are available almost exclusively in organic form.

In the present study, the contributions of organic food to processed meat, fast food or non-alcoholic beverage consumption were among the lowest. A possible explanation for these results might be the fact that such products are mostly unavailable on the market in organic form. Besides, these products may not be considered “healthy” and therefore are not the organic consumers’ preferred choices as observed before in French adults [25].

The key strengths and original aspects of this study were its large sample size and the innovative approach in the assessment of organic food consumption focusing on absolute and relative shares of organic food in the diet. The large sample size ensures capturing large variations in dietary behaviours and in the amount of organic foods consumed. A further strength of the current study was the use of a semi-quantitative food frequency questionnaire with over 260 items including a very large range of foods, which enabled making a reliable estimation of usual diet over the previous year.

However, several limitations to this study should be mentioned. The contribution of organic food to the diet in the present study seems high regarding its share in the food market [24]. Several hypothesizes may explain such figures.

Firstly, the participants enrolled in our study were volunteers in a nutrition cohort and were probably more interested in nutritional issues and healthy lifestyles including organic food issues than the general population. The participants of the NutriNet-Santé cohort exhibit particular characteristics when compared to the general French population [49]. They are more often women and more often a holder of a university degree. This has led to some self-selection (or recruitment) biases. To partly overcome this limitation, all analyses were weighted for each gender according to age, occupational category, area of residence and whether or not the household included at least one child (<18 years) using the iterative proportional fitting procedure according to national census [41] in order to make our sample more representative socio-demographically of the French population. Nevertheless, the nutrition interest of the subjects of the cohort still remains.

Furthermore, individuals selected in the final sample exhibited particular characteristics when compared to other individuals of the cohort. They were older and more often men. As the questionnaire was optional, organic food consumers were certainly more willing to fill out this questionnaire than non-consumers.

In addition to this recruitment bias, a social desirability bias may have occurred as reported in other work [50]. It has been shown that social desirability traits may influence self-reported dietary measures and, in turn, organic food consumption has been probably overestimated. However, a validation study comparing the same food frequency questionnaire with repeated 24 h records exhibited acceptable relative validity and good reproducibility [38] although the question relating to organic food consumption frequency has not been validated.

Moreover, this high consumption of organic food must be interpreted in the light of the use of the ordinal scale: only five choices were given to participants. Thus, a percentage of 25% was allocated to the frequency “rarely”, which does not reflect the very occasional consumers. Nevertheless, in a sensitive analysis, we attributed a percentage of 10% to the frequency rarely and the results were not substantially modified. Similarly, when using Monte-Carlo simulations, with the modalities which were not set-values but were allowed to vary along a uniform distribution, results remained almost unchanged. Finally, data collection is based on self-reported questionnaires which are prone to measurement errors.

Caution is therefore needed when extrapolating the results to the consumption of the general population. The findings are estimates of the contribution of organic food in the diet using a specific tool in a particular population calculated from self-reported consumption. However, our study provides a particularly original contribution to the literature as there remains a paucity of data concerning the contribution of organic food in the diet. Besides, our findings seem consistent with the current food market in terms of the food groups that are the largest organic contributors.

Additionally, to determine whether participants knew what *organic* referred to, we tested knowledge of official organic labels through a dedicated questionnaire (data not shown). Among those who responded to both questionnaires (*N* = 23,010), 93% were able to identify the French organic label “AB”.

## 5. Conclusions

To conclude, this study is original in its innovative approach, with its focus on organic food consumption as a whole and by food group, and in terms of frequency and of absolute and relative intake. We showed that, for some food groups, organic food consumption was not marginal in the diet of our study population. Overall, organic fruits, vegetables and related products were integral components of the diet. Also, organic eggs were quite widely consumed; however, lesser quantities of organic meat and meat products were consumed. Organic fast food, processed food or sweetened foods had lower contributions and efforts in that regard should be made. Organic food consumers are a very large and heterogeneous group and more research is needed to better characterize the diet of non-, occasional, and regular organic food consumers. Further research should accurately investigate the specificities of such consumers to shed further light on the potential relationships between the level of organic food consumption and health.

## Supplementary Materials

Supplementary materials can be accessed at: http://www.mdpi.com/2072-6643/7/10/5417/s1.
