# Supplementary material for: Contribution of Organic Food to the Diet in a Large Sample of French Adults (the NutriNet-Santé Cohort Study)
Source: Nutrients. 2015 Oct 21;7(10):8615–32. doi: 10.3390/nu7105417 (PMC4632437; doi:10.3390/nu7105417)
Supplement: Supplementary file 1 [file nutrients-07-05417-s001.docx]

Supplemental Information

**Table S1.** Percentage of organic food consumers by food group, NutriNet-Santé Study, N = 28,245.

|  | **All Individuals (%)** | | | **Organic Food Consumers * (%)** | | |
| --- | --- | --- | --- | --- | --- | --- |
|  | **(*n* = 28,245)** | | | **Non-organic consumers include 8.4% of women and 14.7% of men *i.e.*, 11.4% of the whole sample** | | |
|  | **% of subjects who consume the food group** | **% of consumers with at least 50% of this food group from organic origin** | **% of consumers with 100% of this food group from organic origin** | **% of subjects who consume the food group** | **% of consumers with at least 50% of this food group from organic origin** | **% of consumers with 100% of this food group from organic origin** |
| Vegetables | 99.9 | 27.2 | 3.2 | 99.9 | 30.7 | 3.6 |
| Soup | 85.5 | 26.7 | 12.5 | 88.0 | 29.3 | 13.7 |
| Fruits | 98.9 | 24.4 | 3.3 | 98.9 | 27.5 | 3.7 |
| Fruit juice | 76.8 | 24.2 | 9.4 | 78.4 | 26.8 | 10.4 |
| Nuts | 64.7 | 28.6 | 15.6 | 68.7 | 30.5 | 16.6 |
| Meat | 92.1 | 14.0 | 1.5 | 91.6 | 15.9 | 1.8 |
| Processed meat | 90.7 | 9.9 | 1.1 | 90.9 | 11.1 | 1.2 |
| Fish | 90.5 | 9.5 | 1.0 | 90.6 | 10.7 | 1.1 |
| Poultry | 92.0 | 18.1 | 6.7 | 91.6 | 20.6 | 7.6 |
| Eggs | 93.6 | 41.7 | 25.6 | 93.6 | 47.1 | 28.9 |
| Milk | 35.6 | 24.4 | 13.6 | 35.2 | 27.8 | 15.6 |
| Dairy products | 89.4 | 18.7 | 6.3 | 89.7 | 21.1 | 7.1 |
| Cheese | 94.3 | 10.1 | 1.4 | 94.6 | 11.3 | 1.6 |
| Milky desserts | 63.7 | 7.9 | 2.7 | 64.5 | 8.8 | 3.0 |
| Potatoes | 98.9 | 24.1 | 6.4 | 99.1 | 27.1 | 7.2 |
| Bread | 84.7 | 8.3 | 2.7 | 83.9 | 9.4 | 3.0 |
| Cereals ^†^ | 99.3 | 22.2 | 6.7 | 99.3 | 25.1 | 7.5 |
| Wholegrain products ^‡^ | 73.3 | 33.6 | 11.3 | 77.0 | 36.1 | 12.1 |
| Oil | 96.9 | 32.3 | 12.7 | 98.1 | 36.1 | 14.2 |
| Butter/Margarine | 90.0 | 19.5 | 10.1 | 90.4 | 21.9 | 11.4 |
| Cookies | 87.5 | 9.9 | 3.1 | 87.9 | 11.1 | 3.4 |
| Nonalcoholic drinks ^§^ | 100.0 | 3.4 | 0.0 | 100.0 | 3.8 | 0.0 |
| Sweet | 99.0 | 19.5 | 1.5 | 99.7 | 21.9 | 1.7 |
| Fast food | 95.5 | 11.2 | 1.9 | 96.0 | 12.5 | 2.1 |
| Meat substitutes | 27.4 | 79.0 | 58.1 | 30.3 | 80.5 | 59.1 |
| Dressing | 95.7 | 17.5 | 4.6 | 96.3 | 19.6 | 5.1 |
| Alcohol | 87.1 | 8.0 | 1.1 | 88.6 | 8.9 | 1.2 |

**Table S1.** *Cont*.

|  | **All Individuals (%)** | | | **Organic Food Consumers * (%)** | | |
| --- | --- | --- | --- | --- | --- | --- |
|  | **(*n* = 28,245)** | | | **Non-organic consumers include 8.4% of women and 14.7% of men *i.e.*, 11.4% of the whole sample** | | |
|  | **% of subjects who consume the food group** | **% of consumers with at least 50% of this food group from organic origin** | **% of consumers with 100% of this food group from organic origin** | **% of subjects who consume the food group** | **% of consumers with at least 50% of this food group from organic origin** | **% of consumers with 100% of this food group from organic origin** |
| Snacks | 90.9 | 14.3 | 3.4 | 91.9 | 16.0 | 3.8 |
| Grains | 36.2 | 68.4 | 44.1 | 39.5 | 70.6 | 45.6 |
| Other fats **^\|^**^\|^ | 89.5 | 20.1 | 8.0 | 91.3 | 22.3 | 8.9 |
| Dairy substitutes ^¶^ | 24.3 | 62.5 | 40.1 | 26.5 | 64.6 | 41.4 |
| Legumes | 83.8 | 23.7 | 13.6 | 86.8 | 25.9 | 14.8 |
| Soda | 72.0 | 7.3 | 2.8 | 71.7 | 8.3 | 3.2 |

* Individuals who report consuming at least one organic food item; ^†^ Including pasta, white rice, muesli, semolina and breakfast cereals; ^‡^ Including wholegrain bread, wholegrain rice and wholegrain pasta; ^§^ Including coffee, tea, chicory, hot chocolate and water; **^|^**^|^ Including mayonnaise, fresh cream, vegetal fresh cream;
^¶^ Including soy yogurt, vegetal-based cheese, vegan fresh cheese, soy milk.

**Table S2.** Comparison between shares of organic food consumption in the diet and by food group across gender (1) by allocating a fixed percentage of 25% to the modality rarely; (2) by allocating a fixed percentage of 10% to the modality rarely and (3) by using Monte-Carlo simulations, NutriNet-Santé Study, N = 28,245.

|  | **Women** | | | | | | **Men** | | | | | |
| --- | --- | --- | --- | --- | --- | --- | --- | --- | --- | --- | --- | --- |
|  | ***n* = 20,080** | | | | | | ***n* = 7265** | | | | | |
|  | **Allocation of 25% to the modality rarely** | | **Allocation of 10% to the modality rarely** | | **Monte Carlo simulations *** | | **Allocation of 25% to the modality rarely** | | **Allocation of 10% to the modality rarely** | | **Monte Carlo simulations *** | |
|  | **Mean** | **SD** | **Mean** | **SD** | **Mean** | **SD** | **Mean** | **SD** | **Mean** | **SD** | **Mean** | **SD** |
| **Total intake** | 0.20 | 0.18 | 0.18 | 0.18 | 0.19 | 0.17 | 0.18 | 0.28 | 0.16 | 0.27 | 0.17 | 0.27 |
| **Food groups** |  |  |  |  |  |  |  |  |  |  |  |  |
| Vegetables | 0.31 | 0.27 | 0.28 | 0.28 | 0.30 | 0.26 | 0.28 | 0.44 | 0.26 | 0.45 | 0.28 | 0.43 |
| Soup | 0.34 | 0.30 | 0.32 | 0.31 | 0.33 | 0.30 | 0.33 | 0.46 | 0.30 | 0.48 | 0.30 | 0.26 |
| Fruits | 0.29 | 0.26 | 0.26 | 0.26 | 0.28 | 0.25 | 0.28 | 0.44 | 0.25 | 0.44 | 0.27 | 0.43 |
| Fruit juice | 0.33 | 0.28 | 0.30 | 0.29 | 0.32 | 0.28 | 0.29 | 0.46 | 0.26 | 0.47 | 0.28 | 0.46 |
| Nuts | 0.35 | 0.30 | 0.32 | 0.31 | 0.34 | 0.30 | 0.33 | 0.48 | 0.30 | 0.49 | 0.33 | 0.47 |
| Meat | 0.18 | 0.22 | 0.15 | 0.21 | 0.18 | 0.21 | 0.18 | 0.35 | 0.15 | 0.34 | 0.18 | 0.34 |
| Processed meat | 0.15 | 0.19 | 0.12 | 0.19 | 0.15 | 0.19 | 0.16 | 0.32 | 0.13 | 0.31 | 0.16 | 0.31 |
| Fish | 0.15 | 0.19 | 0.11 | 0.18 | 0.14 | 0.18 | 0.16 | 0.33 | 0.13 | 0.32 | 0.15 | 0.31 |
| Poultry | 0.27 | 0.26 | 0.24 | 0.27 | 0.25 | 0.42 | 0.26 | 0.42 | 0.22 | 0.43 | 0.25 | 0.42 |
| Eggs | 0.52 | 0.34 | 0.50 | 0.35 | 0.50 | 0.33 | 0.40 | 0.54 | 0.38 | 0.56 | 0.39 | 0.52 |
| Milk | 0.29 | 0.32 | 0.26 | 0.33 | 0.28 | 0.31 | 0.28 | 0.56 | 0.26 | 0.57 | 0.28 | 0.55 |
| Dairy products | 0.24 | 0.27 | 0.21 | 0.27 | 0.23 | 0.26 | 0.23 | 0.44 | 0.20 | 0.44 | 0.22 | 0.42 |
| Cheese | 0.15 | 0.20 | 0.12 | 0.20 | 0.16 | 0.32 | 0.17 | 0.33 | 0.14 | 0.33 | 0.16 | 0.32 |

**Table S2.** *Cont*.

|  | **Women** | | | | | | **Men** | | | | | |
| --- | --- | --- | --- | --- | --- | --- | --- | --- | --- | --- | --- | --- |
|  | ***n* = 20,080** | | | | | | ***n* = 7265** | | | | | |
|  | **Allocation of 25% to the modality rarely** | | **Allocation of 10% to the modality rarely** | | **Monte Carlo simulations *** | | **Allocation of 25% to the modality rarely** | | **Allocation of 10% to the modality rarely** | | **Monte Carlo simulations *** | |
|  | **Mean** | **SD** | **Mean** | **SD** | **Mean** | **SD** | **Mean** | **SD** | **Mean** | **SD** | **Mean** | **SD** |
| Milky desserts | 0.14 | 0.21 | 0.11 | 0.20 | 0.14 | 0.20 | 0.12 | 0.30 | 0.10 | 0.29 | 0.12 | 0.29 |
| Potatoes | 0.28 | 0.30 | 0.26 | 0.30 | 0.27 | 0.29 | 0.26 | 0.46 | 0.23 | 0.46 | 0.26 | 0.44 |
| Bread | 0.16 | 0.21 | 0.12 | 0.20 | 0.15 | 0.20 | 0.16 | 0.34 | 0.12 | 0.33 | 0.15 | 0.33 |
| Cereals | 0.28 | 0.29 | 0.25 | 0.29 | 0.27 | 0.28 | 0.24 | 0.45 | 0.22 | 0.45 | 0.15 | 0.20 |
| Wholegrain products | 0.37 | 0.30 | 0.33 | 0.31 | 0.35 | 0.48 | 0.37 | 0.48 | 0.33 | 0.50 | 0.35 | 0.48 |
| Oil | 0.36 | 0.32 | 0.34 | 0.33 | 0.35 | 0.31 | 0.33 | 0.51 | 0.31 | 0.52 | 0.32 | 0.49 |
| Butter/Margarine | 0.25 | 0.29 | 0.22 | 0.29 | 0.24 | 0.28 | 0.24 | 0.46 | 0.21 | 0.47 | 0.23 | 0.45 |
| Cookies | 0.15 | 0.22 | 0.13 | 0.22 | 0.15 | 0.21 | 0.13 | 0.31 | 0.10 | 0.30 | 0.13 | 0.30 |
| Nonalcoholic drinks | 0.11 | 0.14 | 0.10 | 0.14 | 0.10 | 0.13 | 0.09 | 0.20 | 0.08 | 0.20 | 0.08 | 0.20 |
| Sweet | 0.25 | 0.24 | 0.23 | 0.24 | 0.25 | 0.23 | 0.24 | 0.38 | 0.22 | 0.38 | 0.23 | 0.36 |
| Fast food | 0.17 | 0.22 | 0.15 | 0.22 | 0.17 | 0.21 | 0.15 | 0.32 | 0.12 | 0.31 | 0.15 | 0.31 |
| Meat substitutes | 0.81 | 0.26 | 0.80 | 0.27 | 0.78 | 0.25 | 0.76 | 0.52 | 0.75 | 0.54 | 0.72 | 0.50 |
| Dressing | 0.22 | 0.26 | 0.20 | 0.26 | 0.22 | 0.25 | 0.20 | 0.39 | 0.18 | 0.39 | 0.20 | 0.38 |
| Alcohol | 0.14 | 0.18 | 0.11 | 0.17 | 0.13 | 0.17 | 0.16 | 0.31 | 0.12 | 0.30 | 0.15 | 0.30 |
| Snacks | 0.19 | 0.25 | 0.17 | 0.25 | 0.19 | 0.24 | 0.17 | 0.37 | 0.14 | 0.37 | 0.16 | 0.36 |
| Grains | 0.70 | 0.31 | 0.68 | 0.32 | 0.67 | 0.29 | 0.64 | 0.55 | 0.62 | 0.58 | 0.61 | 0.54 |
| Other fats | 0.25 | 0.29 | 0.23 | 0.29 | 0.24 | 0.28 | 0.23 | 0.43 | 0.21 | 0.44 | 0.23 | 0.42 |
| Dairy substitutes | 0.63 | 0.33 | 0.61 | 0.34 | 0.60 | 0.32 | 0.65 | 0.58 | 0.63 | 0.60 | 0.62 | 0.55 |
| Legumes | 0.31 | 0.31 | 0.28 | 0.31 | 0.30 | 0.30 | 0.28 | 0.47 | 0.26 | 0.48 | 0.28 | 0.46 |
| Soda | 0.11 | 0.20 | 0.10 | 0.19 | 0.11 | 0.19 | 0.13 | 0.36 | 0.11 | 0.35 | 0.13 | 0.33 |

* 20 simulations were performed.

**Table S3.** Consumption of organic food (g/day) according to several sociodemographic and lifestyle factors, NutriNet-Santé Study, *N* = 28,245.

| **Food Groups (g/day)** | **≤Median Age (48 years old)** | | **>Median Age (48 years old)** | | **<High School Diploma** | | **≥High School Diploma** | | **Income < 1800 Euros ^¶^** | | **Income ≥ 1800 Euros ^¶^** | |
| --- | --- | --- | --- | --- | --- | --- | --- | --- | --- | --- | --- | --- |
|  | **(*n* = 9743)** | | **(*n* = 18,502)** | | **(*n* = 10,470)** | | **(*n* = 17,775)** | | **(*n* = 9275)** | | **(*n* = 15,493)** | |
|  | **Total** | **Organic** | **Total** | **Organic** | **Total** | **Organic** | **Total** | **Organic** | **Total** | **Organic** | **Total** | **Organic** |
|  | **Mean ± SD** | **Mean ± SD** | **Mean ± SD** | **Mean ± SD** | **Mean ± SD** | **Mean ± SD** | **Mean ± SD** | **Mean ± SD** | **Mean ± SD** | **Mean ± SD** | **Mean ± SD** | **Mean ± SD** |
| Total Intake | 3415.25 ± 1430.5 | 631.74 ± 896.24 | 3486.20 ± 1018.2 | 689.27 ± 697.32 | 3482.64 ± 1736.1 | 637.17 ± 1106.6 | 3354.14 ± 649.97 | 730.70 ± 473.07 | 3496.49 ± 1483.7 | 588.44 ± 903.85 | 3462.08 ± 931.63 | 765.39 ± 669.99 |
| Vegetables | 255.55 ± 280.79 | 97.57 ± 223.67 | 277.88 ± 158.49 | 94.33 ± 125.84 | 274.00 ± 306.34 | 95.95 ± 246.47 | 244s.63 ± 118.45 | 95.96 ± 90.16 | 259.81 ± 248.31 | 82.34 ± 180.47 | 278.95 ± 169.78 | 113.70 ± 156.75 |
| Soup | 45.63 ± 92.86 | 19.15 ± 61.43 | 94.63 ± 99.74 | 34.94 ± 62.05 | 74.20 ± 150.24 | 27.57 ± 90.17 | 57.65 ± 51.57 | 25.42 ± 37.20 | 69.52 ± 128.73 | 24.08 ± 73.89 | 75.15 ± 79.72 | 33.36 ± 55.45 |
| Fruits | 235.88 ± 350.12 | 78.01 ± 204.77 | 336.99 ± 280.30 | 98.33 ± 153.46 | 307.95 ± 467.32 | 90.14 ± 247.57 | 221.10 ± 148.85 | 82.15 ± 07.42 | 281.68 ± 406.06 | 78.82 ± 211.79 | 291.67 ± 232.34 | 102.80 ± 147.36 |
| Fruit juice | 94.69 ± 143.66 | 31.05 ± 81.88 | 77.20 ± 97.67 | 25.19 ± 53.27 | 80.50 ± 162.09 | 24.19 ± 83.31 | 102.43 ± 75.80 | 39.99 ± 49.79 | 84.88 ± 143.76 | 23.89 ± 77.09 | 85.66 ± 92.59 | 30.99 ± 52.87 |
| Nuts | 2.29 ± 7.09 | 1.35 ± 5.62 | 3.78 ± 7.00 | 1.61 ± 4.41 | 3.18 ± 10.44 | 1.51 ± 7.18 | 2.59 ± 3.89 | 1.41 ± 2.68 | 2.82 ± 8.69 | 1.32 ± 6.10 | 3.20 ± 5.32 | 1.55 ± 3.59 |
| Meat | 72.56 ± 122.83 | 10.05 ± 25.45 | 71.97 ± 55.94 | 13.48 ± 24.44 | 76.08 ± 131.72 | 11.89 ± 36.69 | 60.74 ± 35.17 | 11.37 ± 13.73 | 75.61 ± 122.84 | 10.26 ± 30.69 | 69.04 ± 53.85 | 13.58 ± 20.34 |
| Processed meat | 36.55 ± 37.89 | 4.93 ± 12.28 | 32.47 ± 29.28 | 5.50 ± 11.93 | 34.41 ± 48.09 | 5.00 ± 17.44 | 34.84 ± 17.99 | 5.86 ± 7.19 | 35.86 ± 39.67 | 4.29 ± 12.79 | 33.19 ± 26.25 | 6.45 ± 11.68 |
| Fish | 33.84 ± 52.15 | 5.53 ± 20.69 | 48.55 ± 39.94 | 8.18 ± 15.49 | 42.11 ± 65.18 | 6.68 ± 23.97 | 38.36 ± 27.03 | 7.37 ± 12.18 | 37.50 ± 50.37 | 6.17 ± 20.40 | 47.57 ± 40.48 | 7.84 ± 15.62 |
| Poultry | 28.09 ± 39.02 | 5.72 ± 13.13 | 23.08 ± 22.65 | 6.09 ± 9.30 | 25.95 ± 42.72 | 5.91 ± 15.25 | 24.50 ± 17.40 | 5.89 ± 6.90 | 27.02 ± 33.92 | 5.23 ± 12.61 | 25.73 ± 25.96 | 7.09 ± 9.36 |
| Eggs | 12.87 ± 33.77 | 5.20 ± 13.11 | 12.57 ± 12.62 | 6.15 ± 7.90 | 12.24 ± 21.23 | 5.76 ± 14.83 | 14.16 ± 22.90 | 5.43 ± 5.48 | 13.88 ± 35.12 | 5.13 ± 10.95 | 12.01 ± 11.70 | 6.64 ± 9.61 |
| Milk | 74.53 ± 178.49 | 18.02 ± 85.26 | 56.57 ± 118.18 | 14.49 ± 53.36 | 64.08 ± 199.11 | 14.59 ± 92.33 | 70.06 ± 93.63 | 21.30 ± 43.80 | 69.69 ± 177.70 | 14.56 ± 78.59 | 60.61 ± 110.20 | 18.12 ± 52.01 |
| Dairy products | 139.18 ± 170.69 | 21.73 ± 53.99 | 159.86 ± 125.25 | 38.51 ± 64.17 | 155.76 ± 210.55 | 29.50 ± 90.54 | 130.62 ± 78.53 | 31.90 ± 34.15 | 155.43 ± 183.08 | 27.80 ± 77.97 | 139.09 ± 108.36 | 33.47 ± 47.97 |
| Cheese | 37.68 ± 52.50 | 5.44 ± 15.99 | 44.75 ± 40.99 | 7.23 ± 14.47 | 41.40 ± 64.58 | 5.78 ± 20.63 | 40.61 ± 28.68 | 7.98 ± 10.35 | 42.39 ± 55.05 | 5.68 ± 17.20 | 42.71 ± 38.02 | 7.75 ± 13.88 |
| Milky desserts | 12.74 ± 31.63 | 1.03 ± 5.84 | 11.26 ± 21.85 | 1.66 ± 7.76 | 12.04 ± 37.60 | 1.32 ± 10.73 | 11.89 ± 14.61 | 1.41 ± 3.70 | 13.06 ± 33.65 | 1.14 ± 6.67 | 11.09 ± 19.48 | 1.53 ± 7.47 |
| Potatoes | 28.97 ± 54.96 | 7.29 ± 16.44 | 27.12 ± 23.51 | 7.11 ± 12.42 | 29.77 ± 58.91 | 7.16 ± 20.42 | 22.85 ± 13.17 | 7.35 ± 7.96 | 31.91 ± 58.59 | 6.88 ± 17.55 | 23.61 ± 17.81 | 7.18 ± 10.13 |
| Bread | 57.79 ± 70.63 | 7.48 ± 21.68 | 55.53 ± 52.25 | 7.61 ± 18.39 | 55.99 ± 83.66 | 7.00 ± 28.74 | 58.68 ± 38.12 | 9.17 ± 11.04 | 59.53 ± 72.68 | 7.02 ± 23.18 | 52.86 ± 45.74 | 7.76 ± 15.75 |
| Cereals* | 113.98 ± 123.52 | 28.06 ± 61.35 | 74.73 ± 60.07 | 17.98 ± 35.32 | 91.33 ± 132.01 | 20.42 ± 63.70 | 103.65 ± 49.21 | 30.93 ± 31.29 | 107.13 ± 126.36 | 23.31 ± 61.10 | 84.38 ± 59.95 | 23.67 ± 35.23 |
| Wholegrain products ^†^ | 53.62 ± 114.77 | 31.23 ± 90.44 | 57.22 ± 65.66 | 24.79 ± 48.94 | 56.24 ± 127.97 | 27.77 ± 97.45 | 52.91 ± 45.43 | 28.79 ± 37.41 | 55.12 ± 120.53 | 28.46 ± 94.75 | 59.08 ± 59.86 | 29.59 ± 45.10 |
| Oil | 16.33 ± 20.65 | 6.57 ± 13.78 | 20.87 ± 16.78 | 7.92 ± 11.06 | 18.89 ± 27.30 | 6.97 ± 16.97 | 17.71 ± 9.79 | 8.07 ± 7.89 | 17.81 ± 20.36 | 6.89 ± 15.55 | 20.05 ± 17.15 | 7.82 ± 9.31 |
| Butter/Margarine | 5.99 ± 7.63 | 1.20 ± 3.43 | 7.28 ± 6.46 | 1.66 ± 3.08 | 6.83 ± 10.00 | 1.30 ± 4.33 | 6.05 ± 4.12 | 1.81 ± 2.30 | 6.82 ± 8.63 | 1.27 ± 3.82 | 6.25 ± 5.59 | 1.57 ± 2.70 |
| Cookies | 15.63 ± 29.52 | 1.32 ± 5.48 | 9.42 ± 14.32 | 1.37 ± 4.12 | 12.92 ± 31.96 | 1.28 ± 6.65 | 11.37 ± 10.20 | 1.54 ± 2.83 | 15.26 ± 30.94 | 1.36 ± 5.60 | 9.90 ± 12.26 | 1.40 ± 4.02 |
| Nonalcoholic drinks ^‡^ | 1674.27 ± 1069.7 | 138.61 ± 301.77 | 1674.48 ± 742.27 | 189.30 ± 276.82 | 1664.65 ± 1277.2 | 149.39 ± 391.34 | 1703.74 ± 489.25 | 207.71 ± 198.70 | 1713.20 ± 1116.7 | 147.26 ± 331.26 | 1652.54 ± 676.89 | 188.12 ± 255.44 |
| Sweet | 50.41 ± 50.38 | 11.19 ± 21.78 | 50.25 ± 38.48 | 12.82 ± 21.50 | 49.88 ± 63.42 | 11.63 ± 31.86 | 51.70 ± 23.72 | 13.13 ± 11.99 | 52.07 ± 54.05 | 11.25 ± 23.60 | 49.93 ± 32.14 | 12.66 ± 14.02 |
| Fast food | 65.26 ± 268.78 | 11.65 ± 67.53 | 29.67 ± 27.75 | 4.72 ± 9.40 | 49.44 ± 261.83 | 8.61 ± 65.65 | 41.63 ± 22.42 | 6.92 ± 8.45 | 37.69 ± 46.18 | 5.42 ± 14.52 | 63.81 ± 211.93 | 12.83 ± 52.88 |
| Meat substitutes | 9.61 ± 34.41 | 8.00 ± 30.41 | 4.63 ± 19.57 | 4.27 ± 19.05 | 7.71 ± 38.84 | 6.55 ± 35.43 | 5.38 ± 12.93 | 4.89 ± 12.23 | 6.49 ± 29.91 | 5.42 ± 26.82 | 7.79 ± 22.76 | 6.84 ± 21.48 |
| Dressing | 7.72 ± 10.55 | 1.45 ± 4.68 | 7.09 ± 7.21 | 1.47 ± 3.62 | 7.22 ± 11.46 | 1.37 ± 5.56 | 7.96 ± 6.15 | 1.74 ± 2.72 | 7.42 ± 9.81 | 1.36 ± 4.96 | 7.42 ± 6.99 | 1.57 ± 2.84 |
| Alcohol | 68.13 ± 145.32 | 9.96 ± 34.58 | 124.87 ± 152.22 | 19.62 ± 46.86 | 96.72 ± 217.28 | 14.09 ± 61.35 | 95.61 ± 95.74 | 16.84 ± 27.55 | 83.09 ± 171.54 | 12.00 ± 48.28 | 123.15 ± 145.48 | 20.08 ± 40.75 |
| Snacks | 10.79 ± 21.69 | 3.15 ± 11.92 | 9.10 ± 11.82 | 2.48 ± 6.98 | 9.87 ± 23.90 | 2.62 ± 12.37 | 10.19 ± 8.25 | 3.41 ± 6.20 | 9.48 ± 21.48 | 2.27 ± 9.76 | 10.95 ± 11.54 | 3.80 ± 8.53 |
| Grains | 2.62 ± 9.36 | 2.07 ± 8.03 | 3.28 ± 8.16 | 2.52 ± 7.00 | 3.05 ± 12.61 | 2.34 ± 10.61 | 2.65 ± 4.87 | 2.15 ± 4.49 | 2.82 ± 10.43 | 2.08 ± 8.43 | 2.99 ± 7.07 | 2.43 ± 6.47 |
| Other fats ^§^ | 3.91 ± 7.33 | 1.08 ± 3.61 | 2.94 ± 4.33 | 0.76 ± 2.08 | 3.34 ± 8.23 | 0.87 ± 3.84 | 3.69 ± 3.08 | 1.08 ± 1.72 | 3.80 ± 8.02 | 0.94 ± 3.86 | 3.03 ± 3.43 | 0.91 ± 1.75 |
| Dairy substitutes **^\|^**^\|^ | 37.54 ± 137.90 | 32.51 ± 126.76 | 23.76 ± 84.71 | 17.95 ± 77.28 | 31.26 ± 156.70 | 25.39 ± 143.44 | 28.85 ± 59.19 | 24.81 ± 54.68 | 24.32 ± 110.06 | 20.18 ± 98.45 | 39.23 ± 107.11 | 31.83 ± 99.77 |
| Legumes | 26.36 ± 66.42 | 15.86 ± 63.58 | 14.34 ± 25.19 | 5.49 ± 20.65 | 20.95 ± 68.37 | 11.24 ± 64.46 | 18.60 ± 19.53 | 8.99 ± 15.91 | 19.48 ± 42.20 | 9.33 ± 38.20 | 23.29 ± 47.66 | 13.51 ± 45.61 |
| Soda | 84.23 ± 208.74 | 8.26 ± 43.79 | 38.06 ± 92.99 | 3.76 ± 17.34 | 62.68 ± 221.39 | 5.39 ± 39.82 | 56.71 ± 69.65 | 7.90 ± 20.89 | 73.89 ± 209.62 | 5.01 ± 30.62 | 46.15 ± 89.82 | 6.94 ± 23.87 |

**Table S3.** *Cont*.

| **Food Groups (g/day)** | **Rural Area** | | **Urban Area** | | **Physical Activity < 30 min Brisk walking/day ^**^** | | **Physical activity ≥ 30 min Brisk walking/day ^**^** | | **Meat Eaters** | | **Vegetarians and Vegans** | |
| --- | --- | --- | --- | --- | --- | --- | --- | --- | --- | --- | --- | --- |
|  | **(*n* =6235 )** | | **(*n* =22,010 )** | | **(*n* = 5434)** | | **(*n* =19,830)** | | **(*n* = 27,369)** | | **(*n* = 876)** | |
|  | **Total** | **Organic** | **Total** | **Organic** | **Total** | **Organic** | **Total** | **Organic** | **Total** | **Organic** | **Total** | **Organic** |
|  | **Mean ± SD** | **Mean ± SD** | **Mean ± SD** | **Mean ± SD** | **Mean ± SD** | **Mean ± SD** | **Mean ± SD** | **Mean ± SD** | **Mean ± SD** | **Mean ± SD** | **Mean ± SD** | **Mean ± SD** |
| Total Intake | 3446.30 ± 1137.5 | 703.04 ± 845.31 | 3452.14 ± 1188.4 | 645.91 ± 749.78 | 3238.97 ± 1145.8 | 477.50 ± 642.02 | 3494.68 ± 1107.8 | 757.44 ± 782.91 | 3450.34 ± 1175.2 | 605.32 ± 715.71 | 3456.16 ± 1244.1 | 1638.71 ± 1220.2 |
| Vegetables | 267.53 ± 229.50 | 106.34 ± 169.18 | 266.41 ± 203.11 | 92.41 ± 165.24 | 228.88 ± 193.24 | 64.92 ± 117.81 | 279.44 ± 201.41 | 113.00 ± 179.64 | 252.60 ± 181.77 | 79.62 ± 124.34 | 516.70 ± 515.59 | 385.83 ± 505.49 |
| Soup | 75.73 ± 118.78 | 32.52 ± 76.29 | 68.15 ± 94.55 | 25.16 ± 57.66 | 63.87 ± 91.50 | 20.19 ± 51.70 | 74.15 ± 100.09 | 30.49 ± 64.25 | 70.30 ± 100.06 | 25.60 ± 60.32 | 66.13 ± 112.13 | 52.46 ± 102.31 |
| Fruits | 267.80 ± 329.63 | 88.14 ± 169.00 | 292.65 ± 304.40 | 88.15 ± 174.35 | 241.34 ± 259.52 | 59.80 ± 129.57 | 288.03 ± 285.27 | 100.10 ± 175.72 | 282.83 ± 306.20 | 78.80 ± 157.49 | 348.48 ± 410.96 | 254.14 ± 377.09 |
| Fruit juice | 82.01 ± 121.58 | 29.15 ± 70.70 | 87.31 ± 114.28 | 27.77 ± 62.83 | 73.99 ± 108.65 | 18.30 ± 45.44 | 90.73 ± 115.82 | 32.94 ± 69.33 | 84.92 ± 113.83 | 25.87 ± 61.12 | 104.39 ± 167.45 | 68.15 ± 123.17 |
| Nuts | 2.95 ± 7.22 | 1.55 ± 5.42 | 3.06 ± 7.03 | 1.46 ± 4.69 | 2.13 ± 5.10 | 0.88 ± 2.95 | 3.46 ± 7.45 | 1.80 ± 5.37 | 2.79 ± 6.69 | 1.22 ± 4.34 | 7.31 ± 13.53 | 6.12 ± 11.62 |
| Meat | 80.55 ± 96.26 | 17.01 ± 35.47 | 69.44 ± 81.57 | 9.97 ± 20.59 | 71.66 ± 69.60 | 10.23 ± 21.64 | 70.25 ± 87.56 | 12.54 ± 25.36 | 76.18 ± 84.82 | 12.40 ± 25.08 | 2.74 ± 18.51 | 0.41 ± 4.62 |
| Processed meat | 39.24 ± 37.09 | 6.48 ± 14.35 | 32.90 ± 31.01 | 4.78 ± 11.29 | 35.64 ± 32.92 | 4.88 ± 11.22 | 33.74 ± 32.42 | 5.60 ± 12.45 | 36.31 ± 32.07 | 5.49 ± 12.18 | 2.59 ± 14.55 | 0.30 ± 3.05 |
| Fish | 37.24 ± 39.73 | 6.47 ± 18.27 | 42.52 ± 46.48 | 6.98 ± 17.28 | 36.99 ± 38.12 | 4.49 ± 11.79 | 43.06 ± 44.30 | 8.02 ± 18.97 | 42.71 ± 44.50 | 7.04 ± 17.50 | 13.90 ± 49.67 | 3.60 ± 17.23 |
| Poultry | 23.93 ± 25.17 | 6.21 ± 11.48 | 26.16 ± 30.53 | 5.80 ± 10.57 | 26.67 ± 29.24 | 5.08 ± 10.28 | 24.94 ± 29.35 | 6.12 ± 10.55 | 26.93 ± 29.28 | 6.21 ± 10.84 | 1.87 ± 12.68 | 0.47 ± 4.30 |
| Eggs | 12.89 ± 19.22 | 5.87 ± 9.56 | 12.66 ± 23.11 | 5.61 ± 10.15 | 10.28 ± 10.33 | 4.57 ± 7.47 | 13.81 ± 25.32 | 6.34 ± 10.80 | 13.11 ± 22.41 | 5.72 ± 9.81 | 5.67 ± 16.59 | 4.86 ± 15.30 |
| Milk | 59.92 ± 145.50 | 12.35 ± 55.77 | 67.49 ± 141.19 | 17.60 ± 68.76 | 59.71 ± 137.28 | 10.83 ± 55.00 | 67.04 ± 139.49 | 18.93 ± 68.65 | 68.28 ± 141.86 | 16.88 ± 66.68 | 17.39 ± 137.66 | 5.20 ± 43.97 |
| Dairy products | 145.43 ± 139.59 | 34.39 ± 65.99 | 150.89 ± 143.85 | 28.64 ± 60.00 | 144.39 ± 135.73 | 22.96 ± 51.96 | 144.36 ± 136.28 | 33.58 ± 63.43 | 156.23 ± 141.04 | 31.27 ± 61.69 | 30.02 ± 106.31 | 9.38 ± 44.40 |
| Cheese | 47.54 ± 51.33 | 7.06 ± 16.54 | 39.04 ± 43.41 | 6.08 ± 14.57 | 44.21 ± 45.58 | 5.22 ± 13.47 | 40.17 ± 43.55 | 6.83 ± 15.46 | 42.50 ± 44.97 | 6.40 ± 15.00 | 18.22 ± 48.86 | 5.08 ± 15.99 |
| Milky desserts | 13.08 ± 32.57 | 1.57 ± 7.49 | 11.64 ± 23.32 | 1.26 ± 7.07 | 13.27 ± 26.52 | 1.38 ± 7.13 | 11.11 ± 24.19 | 1.40 ± 7.36 | 12.52 ± 25.89 | 1.37 ± 7.21 | 2.81 ± 11.84 | 0.92 ± 5.51 |
| Potatoes | 32.61 ± 57.30 | 9.51 ± 18.43 | 26.49 ± 29.38 | 6.42 ± 12.29 | 26.52 ± 29.31 | 4.47 ± 10.87 | 26.36 ± 23.38 | 7.87 ± 13.59 | 28.44 ± 37.79 | 6.86 ± 13.69 | 21.02 ± 24.14 | 13.40 ± 18.64 |
| Bread | 65.94 ± 68.27 | 8.28 ± 20.81 | 53.50 ± 56.10 | 7.29 ± 19.22 | 63.24 ± 61.23 | 6.09 ± 17.82 | 55.19 ± 58.11 | 8.50 ± 20.34 | 58.25 ± 58.19 | 7.41 ± 19.09 | 28.55 ± 77.13 | 9.83 ± 31.23 |
| Cereals* | 89.71 ± 83.47 | 23.60 ± 53.39 | 96.00 ± 91.10 | 22.84 ± 44.04 | 95.10 ± 86.99 | 21.98 ± 53.80 | 90.05 ± 76.37 | 25.63 ± 44.17 | 93.58 ± 87.00 | 20.39 ± 40.63 | 108.80 ± 146.67 | 69.99 ± 116.04 |
| Wholegrain products ^†^ | 50.41 ± 82.30 | 24.82 ± 68.22 | 57.12 ± 86.77 | 29.11 ± 65.76 | 40.61 ± 57.84 | 15.10 ± 36.78 | 63.53 ± 92.74 | 34.79 ± 73.80 | 51.68 ± 80.87 | 23.56 ± 57.98 | 121.70 ± 159.36 | 107.14 ± 159.66 |
| Oil | 18.38 ± 16.84 | 7.24 ± 13.54 | 18.67 ± 18.76 | 7.24 ± 11.64 | 17.11 ± 15.86 | 5.62 ± 10.54 | 19.24 ± 17.79 | 8.06 ± 12.32 | 18.54 ± 18.17 | 6.85 ± 11.56 | 19.68 ± 23.23 | 14.20 ± 21.19 |
| Butter/Margarine | 7.57 ± 8.09 | 1.63 ± 3.69 | 6.32 ± 6.52 | 1.36 ± 3.06 | 6.69 ± 7.13 | 1.18 ± 2.90 | 6.41 ± 6.57 | 1.58 ± 3.30 | 6.80 ± 6.88 | 1.38 ± 3.13 | 3.64 ± 6.74 | 2.32 ± 4.93 |
| Cookies | 11.50 ± 20.67 | 1.36 ± 5.18 | 12.88 ± 21.19 | 1.34 ± 4.47 | 12.08 ± 21.75 | 1.13 ± 4.53 | 11.91 ± 19.19 | 1.50 ± 4.61 | 12.81 ± 21.09 | 1.32 ± 4.57 | 7.68 ± 19.90 | 1.87 ± 6.27 |
| Non alcoholic drinks ^‡^ | 1686.15 ± 825.35 | 191.08 ± 338.41 | 1670.36 ± 881.26 | 154.63 ± 269.78 | 1614.89 ± 854.28 | 126.94 ± 263.87 | 1684.16 ± 831.81 | 183.79 ± 287.64 | 1685.20 ± 868.28 | 160.57 ± 283.10 | 1482.33 ± 861.30 | 223.00 ± 376.31 |
| Sweet | 53.26 ± 50.30 | 15.01 ± 32.79 | 49.33 ± 40.59 | 10.98 ± 17.06 | 48.82 ± 41.95 | 9.69 ± 19.36 | 50.19 ± 39.49 | 12.98 ± 17.57 | 50.93 ± 42.89 | 11.39 ± 21.17 | 39.67 ± 42.80 | 22.89 ± 28.98 |
| Fast food | 33.22 ± 30.19 | 5.87 ± 13.12 | 52.37 ± 180.79 | 8.98 ± 45.36 | 35.91 ± 32.96 | 4.04 ± 9.53 | 51.61 ± 189.56 | 10.32 ± 47.76 | 48.85 ± 162.76 | 8.05 ± 41.01 | 23.46 ± 35.11 | 10.64 ± 20.52 |
| Meat substitutes | 7.29 ± 31.54 | 6.82 ± 29.90 | 7.07 ± 23.92 | 5.90 ± 21.57 | 4.72 ± 19.01 | 4.12 ± 17.94 | 8.68 ± 27.21 | 7.45 ± 24.73 | 4.09 ± 19.07 | 3.57 ± 17.84 | 61.00 ± 69.44 | 51.66 ± 65.98 |
| Dressing | 7.30 ± 9.70 | 1.67 ± 5.60 | 7.45 ± 8.15 | 1.39 ± 3.44 | 7.65 ± 9.36 | 1.08 ± 4.08 | 7.19 ± 7.76 | 1.62 ± 3.88 | 7.44 ± 8.24 | 1.35 ± 3.78 | 6.74 ± 14.73 | 3.37 ± 8.22 |
| Alcohol | 106.97 ± 188.40 | 16.12 ± 51.90 | 92.85 ± 140.56 | 14.32 ± 40.52 | 96.29 ± 166.46 | 14.25 ± 42.90 | 99.66 ± 147.12 | 16.37 ± 43.42 | 99.38 ± 152.71 | 14.90 ± 43.10 | 44.38 ± 129.26 | 12.54 ± 48.99 |
| Snacks | 9.98 ± 14.73 | 2.58 ± 8.44 | 9.94 ± 16.28 | 2.89 ± 9.15 | 9.56 ± 13.84 | 1.55 ± 5.21 | 10.27 ± 13.98 | 3.38 ± 9.47 | 9.32 ± 14.92 | 2.12 ± 7.08 | 21.13 ± 32.03 | 15.02 ± 27.88 |
| Grains | 3.17 ± 10.07 | 2.42 ± 9.05 | 2.88 ± 8.13 | 2.25 ± 6.82 | 2.26 ± 7.38 | 1.53 ± 5.86 | 3.31 ± 8.75 | 2.67 ± 7.73 | 2.60 ± 8.11 | 1.94 ± 6.80 | 9.18 ± 16.02 | 8.61 ± 15.33 |
| Other fats ^§^ | 3.38 ± 5.49 | 0.90 ± 2.75 | 3.44 ± 5.60 | 0.93 ± 2.70 | 3.08 ± 4.55 | 0.66 ± 2.32 | 3.47 ± 5.46 | 1.05 ± 2.86 | 3.31 ± 5.30 | 0.73 ± 2.13 | 5.56 ± 10.76 | 4.38 ± 8.56 |
| Dairy substitutes\|\| | 19.09 ± 92.29 | 14.18 ± 76.74 | 34.61 ± 109.72 | 29.02 ± 102.37 | 19.56 ± 73.12 | 15.06 ± 64.59 | 36.71 ± 115.41 | 31.13 ± 106.81 | 22.86 ± 96.38 | 17.77 ± 87.76 | 169.04 ± 198.98 | 157.88 ± 184.33 |
| Legumes | 16.16 ± 26.03 | 6.58 ± 21.64 | 21.80 ± 48.30 | 12.08 ± 45.19 | 12.56 ± 23.68 | 4.57 ± 17.23 | 24.29 ± 48.71 | 14.05 ± 46.01 | 15.50 ± 27.99 | 5.82 ± 21.59 | 106.63 ± 160.16 | 97.03 ± 163.54 |
| Soda | 68.37 ± 172.91 | 8.22 ± 41.78 | 58.74 ± 136.93 | 5.26 ± 24.73 | 69.29 ± 171.33 | 4.72 ± 24.86 | 58.14 ± 133.24 | 7.03 ± 31.61 | 62.51 ± 146.06 | 5.45 ± 25.60 | 37.74 ± 130.09 | 16.07 ± 84.76 |

* Including pasta, white rice, muesli, semolina and breakfast cereals; ^†^ Including wholegrain bread, wholegrain rice and wholegrain pasta; ^‡^ Including coffee, tea, chicory, hot chocolate and water; ^§^ Including mayonnaise, fresh cream, vegetal fresh cream; ^||^ Including soy yogurt, vegetal-based cheese, vegan fresh cheese, soy milk; ^¶^ As the question was optional, the (not weighted) sample size was 24,768. **As a question was optional the (not weighted) sample size was 25,264.

© 2015 by the authors; licensee MDPI, Basel, Switzerland. This article is an open access article distributed under the terms and conditions of the Creative Commons by Attribution (CC-BY) license (http://creativecommons.org/licenses/by/4.0/).
